# Effect of Massage on treatment of preterm feeding intolerance: Study protocol for a randomized controlled trial

**DOI:** 10.1002/nop2.1733

**Published:** 2023-04-17

**Authors:** Nan Peng, Lizhen Fu, Rui Li, Xiaohua Liang, Qi Lu

**Affiliations:** ^1^ Department of Neonatology Children’s Hospital of Chongqing Medical University Chongqing China; ^2^ National Clinical Research Center for Child Health and Disorders Chongqing China; ^3^ Ministry of Education Key Laboratory of Child Development and Disorders, Chongqing Key Laboratory of Pediatrics Chongqing China; ^4^ Department of Traditional Chinese Medicine Children's Hospital of Chongqing Medical University Chongqing China; ^5^ Department of Clinical Epidemiology and Bioinformatics Children's Hospital of Chongqing Medical University Chongqing China

**Keywords:** feeding intolerance, massage, preterm infants

## Abstract

**Aim:**

To evaluate the effectiveness of massage on treating feeding intolerance (FI).

**Design:**

A randomized, controlled, prospective clinical trial.

**Methods:**

A total of 104 preterm infants whose gestational age between 28 and 34 weeks and birth weight between 1000 and 2000 g with diagnosis of FI were recruited. Participants were stratified by birth weight (1000–1499 g or 1500–2000 g) and randomized to either the intervention group, who will receive 7 days of massage, or the control group. The primary outcome is the time to reach full enteral nutrition. Secondary outcomes include duration of FI, change of body index, length of hospitalization, change of gastric residual volume, abdomen circumference and defecation measurement before and after 7 days of intervention.

**Results:**

Results of this study, which includes index on FI and physical development, have the potential to provide evidence that massage will alleviate symptoms of FI, and contribute to the long‐term positive outcome of preterm infants.

## 1 BACKGROUND

Over 14.9 million babies are born prematurely across the world every year, and the number is still increasing (Heinonen et al., 2016). Due to immature gastrointestinal function, premature newborns especially those born before 34 weeks, are susceptible to feeding intolerance (FI) (Asadi et al., 2019), and its morbidity could achieve 70% (Ng et al., 2019). Currently, there is no universal definition of FI, the widely accepted one is the following: the gastric residual volume (GRV) was 50% greater than the last feeding volume, with abdominal distension or vomiting (Moore & Wilson, 2011).

FI could badly influence newborns' health. During the neonatal period, it may lead to malnutrition, delayed enteral nutrition, prolonged hospitalization, growth restriction and increase the risk of sepsis and serious gastrointestinal complications such as necrotizing enterocolitis (NEC) (Meister et al., 2019; Neu, 2007). Additionally, it may lead to long‐term negative outcomes such as visceral obesity, glucose intolerance, and increased risk of cardiovascular disease, negative neurodevelopmental outcomes in adulthood (Asadi et al., 2019). At present, treatment for FI includes breastfeeding, modifications in feeding regimes, use of probiotics or medicine, but effectiveness and safety of these methods remain controversial (Basu & Smith, 2020; Fanaro, 2012; Sukwuttichai et al., 2021), feeding intolerance (FI) is still a thorny issue affecting the prognosis of preterm infants. So it is important to find an effective way to cure FI, contributing to recovery of gastrointestinal function and improving long‐term health of preterm infants.

In recent years, massage therapy has been frequently used in neonatal wards for the prevention of FI. Studies have shown that massage could prevent gastric retention and abdominal distention in premature infants, and reduce the length of stay. In addition, massage could stimulate the digestive system and the vagus nerve in premature infants, potentially improving their physical growth (Diego et al., 2007; Heinonen et al., 2016; Tekgündüz et al., 2014). However, there is no consensus on the efficacy of massage in the treatment of FI.

## 2 AIM

To determine the efficacy of massage in the therapy of FI, thus providing a new, safe and efficient method for the treatment of preterm FI.

## 3 METHOD

### 3.1 Study setting

This trial is conducted at a large neonatal treatment center of a tertiary‐level children's hospital, which is affiliated with one of the excellent medical universities in the country. It has a 250‐bed neonatal unit with an annual admission rate of about 8000 newborns over the past 2 years, which could assure a large number of research subjects.

### 3.2 Study design

**FIGURE 1:**
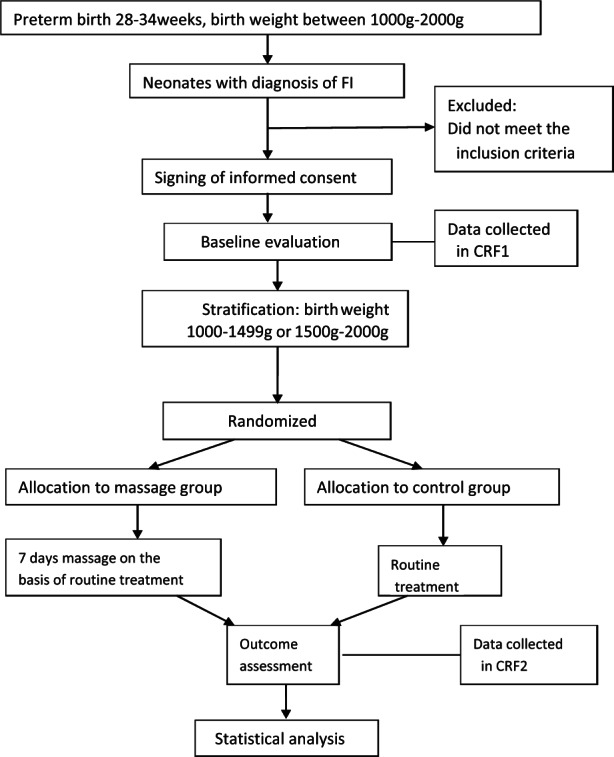
Procedure of study.

Following the Standard Protocol Items: Recommendations for Interventional Trials (Chan et al., 2013), the study protocol describes the design of a randomized, controlled, prospective clinical trial. Preterm infants whose gestational age between 28 and 34 weeks and birth weight between 1000 and 2000 g with the diagnosis of FI were involved in the study. They were stratified by birth weight (1000–1499 g or 1500–2000 g) and randomized to either the intervention group or the control group. Figure 1 shows the procedure of our study.

### 3.3 Participants: Inclusion/exclusion criteria

Table 1 specifies the criteria for inclusion, exclusion, termination and exit criteria. Inclusion and exclusion criteria were defined according to previous literature (Baldassarre et al., 2019; Ng et al., 2018; Yakobson et al., 2020).

**TABLE 1:** Inclusion, exclusion, termination and exit criteria.

*Inclusion criteria*
Meet the FI diagnostic criteria
Admitted to the Neonatal Treatment Center of the Affiliated Children's Hospital of Chongqing Medical University within 24 h after birth
28 weeks ≤ gestational age ≤ 34 weeks, 1000 g ≤ birth weight ≤ 2000 g
In clinically stable condition
The neonate's guardians are willing to participate in this trial
*Exclusion criteria*
Apgar score less than 4 at 5 min
Neonates with skin disease, intestinal obstruction, sepsis, necrotizing enterocolitis, gastrointestinal malformation, congenital heart disease, central nervous system disorder, immune deficiency, or any other disease that may affect the ability to eat and normal growth before recruitment
Neonates who need surgery which requires general anaesthesia (except for ligation of patent ductus arteriosus) before the day of randomization
Neonates whose blood pressure is not stable (using dopamine <5 mg /kg/min is permitted)
Ventilator dependent or FiO_2_ (Fraction of inspired oxygen) > 40% on the day of randomization (continuous positive airway pressure, nasal intubation, oxygen mask are allowed)
Neonates who are diagnosed with Grade III or IV ventricular haemorrhage (IVH) on the day or before randomization
*Termination criteria*
Discharge in a stable condition with doctor's advice
*Exit criteria*
Death or discharge before achieving total enteral nutrition
Guardian request to withdraw from the trail

### 3.4 Sample size calculation

We estimated the sample size by the use of PASS 15. According to a previous study (Kim & Bang, 2018), the average time of achieving total enteral nutrition in the intervention group and control group is 28.3 days and 33.5 days respectively. This study is a superiority test of two independent means, desired power was set at 0.9, *α* was set at 0.025, *Zα* = 1.64, *Zβ* = 0.84. *σ* represents the standard deviation, and δ represents the difference between the mean values of each group. After calculation, 41 participants were needed for each group. Considering a 20% loss rate, 52 participants were needed for each group. A total of 104 participants should be included.

### 3.5 Recruitment and informed consent

During the study period, all the neonates diagnosed with FI were evaluated to see if they fit our criteria. Researchers would introduce this trial to guardians of neonates who meet eligibility criteria in detail. Guardians acknowledged that data about their baby would be collected and used in research, they also acknowledged that they could choose not to participate in this trial and withdraw freely at any time, and this would not impact on their care. When guardians of the neonate agreed to participate in the trial, they were required to sign the informed consent. During the study period, the staff from the quality control department randomly selected the guardians of the subjects and asked them whether they understand the informed consent clearly, to ensure the rights and interests of the subjects and their guardians.

### 3.6 Randomization and blinding

A third‐party personnel created a randomization schedule which was stratified by birth weight (1000–1499 g and 1500–2000 g) using a computer, and then provided it in sealed, opaque, consecutively numbered envelopes. Participants were assigned randomly to one of the two groups by opening the next envelope sequentially from the appropriate set. An independent research assistant responsible for randomization would inform the massager to perform massage when a neonate was assigned to the massage group, and he is the only person aware of which group the neonate had been assigned to. The massager also knows the list of massage groups. Both of them have no access to data of participants and are not responsible for statistical analysis. A researcher who has no access to the allocation group will perform all the assessments for our study.

### 3.7 Variables studied

A researcher who has no access to the allocation group will perform all the assessments for our study. Table 2 shows the demographic variables including maternal characteristics and neonatal characteristics of participants recruited in our trial.

**TABLE 2:** Demographic variables.

Variables	Values
*Maternal characteristics*	
Caesarean delivery	Yes/no
Twin pregnancy	Yes/no
Gestational hypertension	Yes/no
Diabetes during pregnancy	Yes/no
Intrauterine distress	Yes/no
*Neonatal characteristics*	
Gestational age	Weeks
Day of recruitment	Day
Birth weight	g
Boy	Yes/no
Apgar score at 5 min	1–10
Weight at recruitment	g
neonatal critical illness score	Yes/no
Routine treatment received	Antibiotics/caffeine citrate/type of feeding

### 3.8 Intervention

Intervention was provided by a massager (who has rich experience of neonatal nursing and has qualifications for neonatal massage). A 15‐min massage was performed on the massage group twice a day for 7 days. The massage began 30 min before feeding. Before the massage, the massager would thoroughly wash and disinfect hands and warm them. Neonates were placed in the supine position, and their heads and shoulders were pad 30°–45°. All the neonates were under ECG monitoring during massage.

The method of massage, frequency and application time were decided according to expert opinions found in previous studies (Diego et al., 2007; Heinonen et al., 2016; Kim & Bang, 2018; Tekgündüz et al., 2014; Zhu & Gong, 2021). And its biological plausibility and safety have been validated. Massage includes two parts: abdominal massage and skin stroking.

#### 3.8.1 Abdominal massage

- Starting from the infant's base of the ribs, run alternately with four fingers of your two hands vertically downward along both sides of the xiphoid process.
- Starting from the bottom of the ribs, beginning with the xiphoid, your left and right thumbs stroke simultaneously along the lower costal margin.
- Make the ‘I L U’ stroke: tracing ‘I’ down the infant's left costal margin. Then stroke across the infant's abdomen along the base of infant's right rib to his left rib and down, thus trace an inverted ‘L’. Then trace an inverted U. Stroke from the infant's right side, over and around the belly button, then under the left side.
- With the navel as the center of the circle, massage baby's abdomen clockwise.
- Start from the right side of the infant's abdomen, from top to bottom, and press the nine divisions of the abdomen gently.
- Hold the infants' legs with your hands, and make them bent. Then hold the baby's thigh close to the belly, and stay for 3–5 s.

#### 3.8.2 Skin stroking

- Tap 15 times or press gently on the sole of the foot.
- The forearm stroking: stroke baby's skin with your thumb or index finger from the elbow to the wrist stripes at the ulnar side of the forearm, each forearm needs 30‐s stroking. Then use your thumb or index fingers to stroke baby's skin from the wrist to the elbow stripes at the radial side of the forearm, about two 30 s each.

### 3.9 Safety

As safety of massage has been proven in previous studies, our intervention would not induce any complications in most cases. And we took several measurements to avoid the occurrence of adverse events. The strength of massage was controlled so that the baby's skin changes just from pink to white. During massage, the massager paid close attention to the ECG monitor. Massage was suspended when the baby's vital signs (such as respiratory rate, heart rate and oxygen saturation) were not stable (Diego et al., 2009; Indrio et al., 2011), or the baby began to cry, urinate or defecate. Massager would continue massage only when the baby returns to a stable condition.

### 3.10 Outcome

#### 3.10.1 Primary outcome

The time (day) from initiation of enteral nutrition to total enteral nutrition, which is defined as a daily intake of ≥120 mL/Kg/day (Abdallah et al., 2013).

#### 3.10.2 Secondary outcome

1. Days of vomiting, gastric retention (measured by aspirating with a 5‐mL syringe before each feeding) and abdominal distension (defined as a 24‐h abdominal circumference increase of ≥1.5 cm).
2. Weight gain (kg), change of length and head circumference during hospitalization.
3. Length of hospital stay (day).
4. Change of abdomen circumference, gastric retention volume and frequency of defecation (comparison between day 1 and day 7).

Disappearing of vomiting, abdominal distention gastric retention and the amount of milk increased day by day are seemed as alleviation of FI (Lu & Lan, 2020).

### 3.11 Data collection and management

Table 3 shows the schedule of evaluations. Data of each participant were collected on case report forms (CRFs) and then electronically recorded on a computer. CRF1 records participants' baseline information and it was filled in when neonates were included in the trial. CFR2, which includes information about the outcome of this trial, will be filled in during the trial and when neonates discharge with advice.

**TABLE 3:** Schedule of evaluations.

	Staff member	CRF	T1	T2	T3	T4	T5	T6
Symptom of FI	Neonatal assistant	CRF1	**×**					
Informed consent	Research assistant	CRF1	**×**					
Contact information	Research assistant	CRF1	**×**					
Sex	Neonatal physician	CRF1		**×**				
Gestational age	Neonatal physician	CRF1		**×**	**×**			
Mode of delivery	Neonatal physician	CRF1		**×**				
Apgar score	Neonatal physician	CRF1		**×**				
Maternal issues	Neonatal physician	CRF1		**×**				
Weight	Neonatal nurse	CRF2		**×**	**×**			**×**
Gastric residual volume	Neonatal nurse	CRF2				**×**	**×**	
Abdominal circumference	Neonatal nurse	CRF2				**×**	**×**	
Frequency of defecation	Neonatal physician	CRF2				**×**	**×**	
Length	Neonatal nurse	CRF2		**×**				**×**
Head circumference	Neonatal nurse	CRF2		**×**				**×**
Time to achieving total enteral nutrition	Neonatal physician	CRF2						**×**
Duration of abdominal distension	Neonatal physician	CRF2						**×**
Duration of vomiting	Neonatal physician	CRF2						**×**

*Note*: T1, screening; T2, baseline evaluation; T3 randomization; T4, day 1 of intervention; T5, day 7 of intervention; T6, discharge.

Once recorded, data are locked to prevent changes. Access to the data is limited to researchers directly involved with this trial. Data about participants who discontinue or deviate from intervention will not be included in statistical analysis.

### 3.12 Statistical analysis

After the data for the entire sample has been collected, it will be performed using SPSS 25.0 software package. Missing data will be forecasted and filled using multiple imputations. A logistic regression model will be used to adjust for multiple potential confounders plain. Measurement data obeying the normal distribution will be expressed as mean (standard deviation), and t‐test will be used for comparing between two groups. Non‐normally distributed variables will be expressed as median (interquartile range), and compared by the use of non‐parametric Wilcoxon–Mann–Whitney test. Classification data will be described as cases count (construct Proportional) and compared using the chi‐square test or non‐parametric Wilcoxon–Mann–Whitney test. The confidence interval will be set at 95%. *p* < 0.05 will be considered statistically significant.

### 3.13 Validity and reliability

An impartial scientific and administrative Steering Committee will monitor the whole course of the study, the evaluation of new information, patient safety, and protocol adherence. Cronbach's alpha is used to calculate reliability. Cronbach's alpha will be calculated for each dimension to assess the consistency of researchers evaluating neonates' condition. Cronbach's alpha between 0.7 and 0.8 is regarded as acceptable and 0.8 or higher is optimal (Cronbach, 1951).

Data monitoring committee (DMC) is responsible for making sure that researcher performs the recruitment, clinicians document data of participants and trial statisticians are blinded to the allocation group until the results are finalized. The DMC also discusses interim analyses and considers protocol modifications during the study period.

### 3.14 Current study status

The experiment is still ongoing at the time of submission of this paper; however, subject recruitment began in April 2021. Currently, the study has not completed the recruiting phase, and it is anticipated that the experiment will complete in December 2023.

### 3.15 Dissemination of study outcomes

Outcomes will be disseminated through publication and presentations at scientific conferences.

## 4 DISCUSSION

This article describes the methodology of a study designed on the effects of massage on the treatment of preterm FI. A total of 104 preterm infants will be included in this trial, they will be stratified according to their birth weight and then assigned to the massage group or control group randomly. Variables include index on FI and physical development will be recorded.

We combined abdominal massage and skin stroking in our trail. According to scientific literature, abdominal massage stimulates gastrointestinal peristalsis by increasing vagus nerve activity (Moyer et al., 2004). Additionally, it can affect abdominal pressure and have a mechanical and reactive impact on the intestines (Harrington & Haskvitz, 2006; Liu et al., 2005). Skin stroking may activate the baroreceptors and mechanoreceptors of the skin as well as the vagal efferent fibres that innervate the digestive system (Diego et al., 2005; Field, 2016). Consequently, we hypothesized that a combination of abdominal massage and skin stroking may be more effective than earlier interventions.

There were several studies investigating the benefits of massage, but low sample size and differences in the duration and frequency of massage in these studies were their limitations (Ghasemi et al., 2019; Shaeri et al., 2017; Soheila et al., 2013; Zaky Mohamed & Saied Ahmed, 2018). This study with accurate methodology, longer interventions and large sample size has the potential to give proof that preterm infant massage benefits in the recovery of gastrointestinal function and promotes growth and development if it is conducted successfully. Owing to the lack of research on the treatment of FI, it is anticipated that this study will fill a large gap in the literature.

Massage also has other advantages for preterm infants, such as promoting physical development, strengthening the immune system, reducing stress, and reducing the time of hospitalization (Niemi, 2017). So, this study may give a novel nursing practice for enhancing the long‐term outcomes of premature infants and could be widely used in neonatal wards.

There are also some limitations of this study. First, since the research cohort consisted of preterm babies with low birth weight, it is possible that the findings cannot be generalized to the whole newborn community. A second limitation is that participants cannot be made blind. Furthermore, the absence of post‐intervention follow‐up makes it impossible to assess the long‐term prognosis.

## AUTHOR CONTRIBUTIONS

Qi Lu and Nan Peng designed this trial. Lizhen Fu and Rui Li develop the Massage Plan. Nan Peng drafted the manuscript and Qi Lu made contributions to its development. Xiaohua Liang provided consulting on statistical analysis. All authors approved the final manuscript.

## FUNDING INFORMATION

This work study was supported by Chongqing Health Committee and Chongqing Science and Technology Bureau [grant numbers 2021ZY023801].

## CONFLICT OF INTEREST STATEMENT

The authors declare that they have no known competing financial interests or personal relationships that could have appeared to influence the work reported in this paper.

## ETHICS APPROVAL AND CONSENT TO PARTICIPATE

The trial protocol, informed consent forms and other requested documents have been reviewed and approved by the Ethics Committee of Children's Hospital of Chongqing Medical University. We will not begin recruiting in the trial until local ethical approvals have been obtained. The progress and the safety of the study will be accessed every 6 months by the Ethics Committee. Written consent will be obtained from all participants. The trial was registered at the Chinese Clinical Trial Registry (ID: ChiCTR2100045033). Important protocol modifications will be communicated to these agencies. We respect the parents' decisions to join or leave the study at any time.

## CONSENT FOR PUBLICATION

Written informed consent was obtained from the patient/participant for publication of their individual details and accompanying images in this manuscript. The consent form is held by the authors and is available for review by the Editor‐in‐Chief.

## PATIENT OR PUBLIC CONTRIBUTION

Patients involved in this study as participants. Caregivers performed massage therapy and recorded body index of participants in this study.

## TRIAL REGISTRATION

Chinese Clinical Trial Registry, ChiCTR2100045033, Registered on 3 April 2021.

## Data Availability

The data that support the findings of this study are available from the corresponding author upon reasonable request.
